# Neuroprotective effects of ghrelin in cuprizone-induced rat model of multiple sclerosis

**DOI:** 10.1007/s11011-025-01603-z

**Published:** 2025-04-11

**Authors:** Sezai Oruk, Ozlem Ergul Erkec, Zubeyir Huyut, Eda Acikgoz

**Affiliations:** 1 Department of Medical Physiology, Institute of Health Sciences, Van Yuzuncu Yil University, Van, Turkey; 2 Department of Physiology, Faculty of Medicine, Van Yuzuncu Yil University, Van, Turkey; 3 Department of Biochemistry, Faculty of Medicine, Van Yuzuncu Yil University, Van, Turkey; 4 Department of Histology and Embryology, Faculty of Medicine, Van Yuzuncu Yil University, Van, Turkey

**Keywords:** Demyelination, Ghrelin, Inflammation, Neuroprotection, Oxidative stress, Remyelination

## Abstract

Multiple sclerosis (MS) is an inflammatory central nervous system disease characterized by demyelination and axonal loss and is the main cause of non-traumatic neurological disability in young adults. Although there are several treatment approaches to manage the disease, there is no definitive cure for multiple sclerosis. Inflammation and oxidative stress are known to play important roles in the pathophysiology of MS. Ghrelin, a peptide secreted by the stomach, is reported to have neuroprotective properties through several pathways, including attenuating oxidative stress and inflammation. In the present study cuprizone (CPZ)-induced model of MS was used in Wistar albino rats to study the possible anti-inflammatory, antioxidant and neuroprotective effects of ghrelin. Rats were randomly divided into six groups: Control groups (Control_35_ and Control-S_42_), demyelination group, remyelination group, remyelination + ghrelin (20 µg/kg) group and remyelination + ghrelin (40 µg/kg) group. Y maze test was performed on the rats on their last day of the experiment. Oxidative stress and inflammatory parameters were investigated in brain using commercial kits by enzyme-linked immunosorbent assay (ELISA). Luxol fast blue (LFB) and hematoxylen&eosin (H&E) staining were performed in brain tissues. CPZ leads to a significant decrease in glutathione peroxidase (GSH-Px) levels and myelin content and a significant increase in malondialdehyde (MDA), tumor necrosis factor-alpha (TNF-ɑ), interleukin- 6 (IL- 6) levels, the number of lymphatic cells and inflammatory cells. A significant increase in the antioxidant parameter levels and a significant decrease in MDA levels were found in the ghrelin treated groups (*p* < 0.05). CPZ leads to irregular, fragmented, demyelinating nerve fibers. A more significant remyelination was observed in the ghrelin treated groups compared to the other groups (*p* < 0.05). In conclusion, ghrelin treatment showed neuroprotective and antioxidant properties and reduced demyelination in the CPZ-induced rat model of multiple sclerosis.

**Graphical Abstract:**

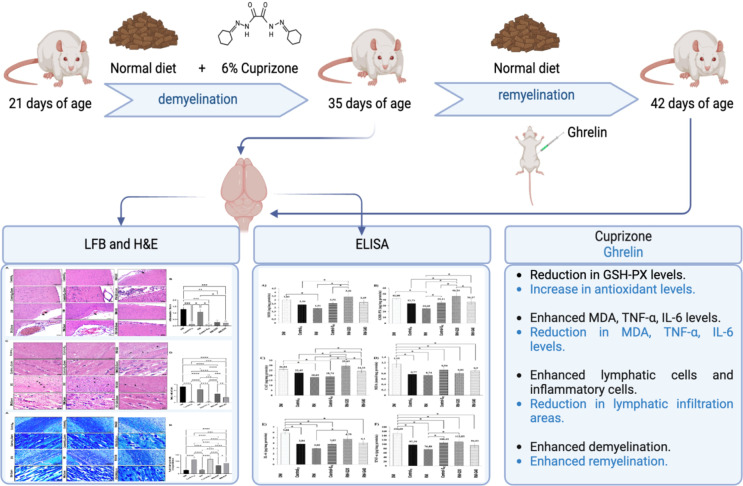

## Introduction

Neurodegenerative diseases are a wide variety of neurological disorders, including multiple sclerosis (MS), Parkinson’s disease, Amyotrophic Lateral Sclerosis and Alzheimer’s disease, characterized by progressive neuronal loss in the nervous system (Wilson et al. 2023; Cantero-Fortiz and Boada 2024). Inflammation and oxidative stress (OS) play an important role in the initiation and progression of neurodegenerative diseases (Zhang et al. 2023; Dash et al. 2024). Therefore, researchers have focused on antioxidant or anti-inflammatory molecules to reduce oxidative stress or inflammation in order to avoid or slow down neurodegenerative diseases such as MS, Parkinson’s disease, Amyotrophic Lateral Sclerosis or Alzheimer’s disease (Goel and Chaudhary 2020; Medhat et al. 2020; Dash et al. 2024; Liang et al. 2024; Muñoz-Jurado et al. 2024).

MS is a chronic inflammatory and demyelinating disease of the central nervous system (CNS) (Oh et al. 2018). This disorder is identified as the major reason of non-traumatic disability among middle-aged and young adults (Dymecka et al. 2021). Currently there is no cure for MS (Dorababu 2025). Therefore, animal models are important for elucidating the complex mechanisms underlying MS and developing promising remyelination therapies (Gharagozloo et al. 2022).

Cuprizone (CPZ)-induced demyelination model is a widely used animal model (Friesen et al. 2024) that mimics the acute, chronic, relapsing and remitting phases of MS (Palumbo and Pellegrini 2017). This toxin-based animal model produces a demyelinating disorder similar to that of in the brains of MS patients (Toomey et al. 2021). CPZ, also called bis (cyclohexanone) oxaldihydrazone, is defined as a copper chelator that effects the activity of Cu^2+^ dependent metalloenzymes including Cu/Zn-superoxide dismutase (SOD), (Martínez-Pinilla et al. 2021). CPZ can cause oxidative stress by inhibiting the activity of antioxidant enzyme Cu/Zn-SOD (Xuan et al. 2015). Oxidative stress has been reported by increased malondialdehyde (MDA) and H_2_O_2_ levels and decreased catalase (CAT) and glutathione peroxidase (GSH-Px) activities (Xuan et al. 2015). As a result of increased reactive oxygen species, cuprizone can damage mitochondrial DNA and membrane, thus causing mitochondrial dysfunction (Xuan et al. 2015). In a previous study it was suggested that, CPZ toxicity may lead to a disruption of enzymes involved in amino-acid metabolism in oligodendrocytes and lead to increased sensitivity to reactive oxygen species (ROS) and energy depletion (Taraboletti et al. 2017). In addition it was reported that cuprizone induces oxidative stress by decreasing antioxidant enzyme levels including SOD (Omotoso et al. 2020), GSH-Px (Omotoso et al. 2020; El-Sayed et al. 2024), glutathione (GSH), CAT (El-Sayed et al. 2024) and increasing brain lipid peroxidation (Omotoso et al. 2020; El-Sayed et al. 2024).

The main effect of CPZ in the CNS is on oligodendrocyte myelination (Toomey et al. 2021). It has been reported that CPZ application led to a decrease in the expression of oligodendrocyte and myelin genes and the number of oligodendrocytes in the corpus callosum, and an increase in the expression of microglial activation genes and the number of microglia in rats (Xavier et al. 2023). CPZ triggers demyelination in the brain cortex (Hashem et al. 2022) and corpus callosum (Moradi et al. 2023). In addition, CPZ leads brain inflammation and OS (Nicola et al. 2024) which are known to play a role in the pathophysiology of MS (Pegoretti et al. 2020). Therefore CPZ model is known as a useful model for new therapeutic approaches to protect oligodendrocytes and induce remyelination (Palumbo and Pellegrini 2017).

Inflammation and OS play important roles in MS (Pegoretti et al. 2020). In pathological circumstances, inflammation and mitochondrial respiratory chain dysfunction trigger increased ROS levels which eventually suppresses natural antioxidant defense system and causes OS (Gonsette 2008). OS, inflammation and excitotoxicity lead neurodegeneration and death of neurons in MS (Woo et al. 2024). Therefore, studies focused on new targets to prevent against inflammation, OS and excitotoxicity.

Ghrelin is a gastrointestinal peptide hormone consisting of 28 amino acids discovered in 1999 (Kojima et al. 1999). Ghrelin is mainly produced in stomach (Mehdar 2021) however also exists different regions of the body including brain (Müller et al. 2015; Erkec et al. 2018). Antioxidant, anti-inflammatory, anti-apoptotic, and neuroprotective effects of ghrelin were reported in CNS disorders including epilepsy, traumatic brain injury, Parkinson and Alzheimer’s disease (Moon et al. 2009; Lopez et al. 2012; Sarlaki et al. 2022; Ergul Erkec et al. 2024a, b). Its inhibitory effects on microglial activity and protective effects on chronic glutamate excitotoxicity were also reported on spinal cord motor neurons (Lim et al. 2011; Lee et al. 2012). Previous studies reported that ghrelin treatment significantly improved inflammatory infiltration and demyelination, reduced mRNA levels or expression of inflammatory cytokines and the clinical severity of the disease in the experimental autoimmune encephalomyelitis (EAE) (Theil et al. 2009; Souza-Moreira et al. 2013; Liu et al. 2019). One of the important pathophysiological mechanisms underlying demyelination and neurodegeneration in MS is OS (Ljubisavljevic 2016; Hollen et al. 2023). However, in our knowledge, the effects of ghrelin treatment on OS have not been investigated in any experimental models of MS. Additionally, the possible anti-inflammatory and neuroprotective effects of ghrelin in the CPZ-induced experimental MS model have not been investigated before. Aim of this study is to evaluate the possible antioxidative, anti-inflammatory and neuroprotective effects of ghrelin in a CPZ-induced rat model of MS.

## Materials and methods

Cuprizone, bis (cyclohexanone) oxaldihidrazone (Cas No: 370 - 81- 0), Hematoxylin (Cas No: 517 - 28- 2), Eosin (Cas No: 17372 - 87- 1), Paraformaldehyde (Cas No: 30525 - 89- 4) were purchased from Sigma- Aldrich (St. Louis, MO, USA). Ghrelin (Cas No: 258338 - 12- 4) was purchased from GenScript (NJ, USA). Luxol Fast Blue (LFB) Stain Kit (Lot: 61230 LBC- 2) was purchased from ScyTek (Logan-Utah USA). ELISA kits: CAT (Cat No: E0869Ra), SOD (Cat No: E0168Ra), GSH-Px (Cat No: E1242Ra), MDA (Cat No: E0156Ra), TNF-ɑ (Cat No: E0764Ra) and IL- 6 (Cat No: E0135Ra) were purchased from BT LAB (Shanghai, China).

### Animals

Forty eight Wistar albino rats (21-days-old, male) were kept in a constant temperature and humidity conditions, in accordance with a twelve-h dark/light cycle and were provided free access to water and food. This study was carried out with an ethical approval from Van Yuzuncu Yil University Ethical Committee (28/05/2020–2020/05–07).

One hemisphere of brain tissue taken from rats was used for ELISA studies and the other hemisphere was used for histological studies. One hemisphere was stored at − 80 °C for ELISA until the day of the study. The other hemisphere was stored in formaldehyde for histopathology until the day of the study.

### Experimental procedure

**Fig. 1:**
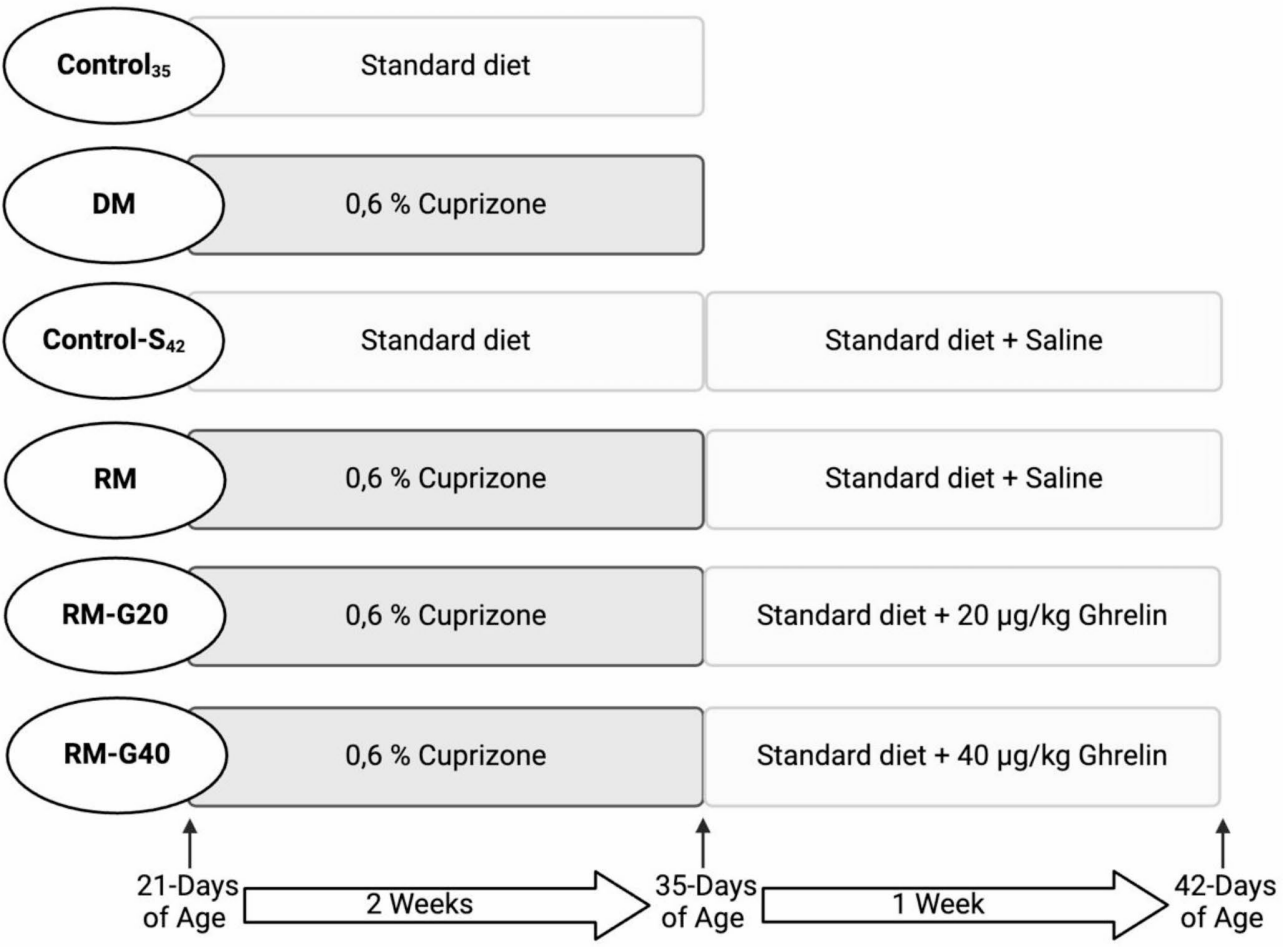
Experimental procedure. (Standard diet: Bayramoglu Feed and Flour Industry Trade Inc., Turkey)

Adamo et al. (2006) first demonstrated that CPZ can be used effectively to induce demyelination in Wistar rats. They reported that demyelination is produced in 21-day-old Wistar rats with CPZ administration in the diet during 2 weeks at a dose of 0.6% and substantially remyelination occurs after 2 weeks on standard diet (Adamo et al. 2006). Therefore, in the present study, animals were weaned at 21 days of age and randomly divided into six groups (*n* = 8). For induction of experimental MS model, demyelination (DM), remyelination (RM), remyelination + ghrelin 20 (RM-G20) and remyelination + ghrelin 40 (RM-G40) groups were fed with a pulverized standard chow pellet supplemented with CPZ (0.6%) that prepared freshly every morning for 2 weeks (Silvestroff et al. 2012). Rats in the control groups (Control_35_ and Control-S_42_) were fed with standard chow diet. Rats in the DM and Control_35_ groups were sacrificed on the 35 days of age. At the age of 35 days, the animals in the other groups were received only the standard diet + saline/ghrelin, no CPZ. Rats were sacrificed on the 42 days of age (Silvestroff et al. 2012). Rats in the RM-G20 and RM-G40 groups were given ghrelin at a dose of 20–40 µg/kg (Ergul Erkec et al. 2024b) respectively and Control-S_42_ was given 1 mL/kg of saline, between the ages of 35 and 42 days for 7 days (Fig. 1).

### Y-maze test

Y-maze test was conducted according to the previous studies at the end of the experiment (for DM and Control_35_ groups 35 days of age and for the other groups 42 days of age) (Ergul Erkec et al. 2024a, b). Briefly, the arms were named A, B and C. The latency of the rats to exit the starting arm, the number of arm entries, and the number of successful changes were recorded for 6 min. The percentage of spontaneous changes was then calculated (Ergul Erkec et al. 2024a, b).

### Tissue preparation

Following the Y-maze test, blood was taken from the apex of the heart by cardiac puncture from anesthetized rats (xylazine 10 mg/kg/ip. and ketamine 50 mg/kg/ip.). Then brain (cerebral hemispheres) was carefully and immediately removed. One hemisphere of the brain was preserved at − 80 °C for ELISA studies and the other hemisphere was preserved in paraformaldehyde (4%, pH: 7.4) for histological studies.

#### Preparation of brain homogenates for ELISA studies

Brain tissues taken from rats were measured on a precision scale and divided into 100 mg pieces and placed in small tubes. 0.9 mL and 50 mM phosphate buffer (pH: 7.4) was added to these tubes (weight/volume = 1/10). A homogenizer was used to liquefy and homogenize the solid brain tissue in this mixture (14 × 5 was mixed again and waited for 10 s). The homogenized tubes were centrifuged at 3000 rpm and 6 ºC for 20 min. After centrifugation, small particles settled to the bottom while the supernatant liquid remained on top. The supernatants were transferred to new eppendorf tubes.

#### Measurement of OS and inflammatory parameters

Protein measurement in the brain homogenates was performed according to the Bradford method (Bradford 1976). CAT, SOD, GSH-Px, MDA, TNF-ɑ and IL- 6 levels in brain homogenates were determined at 450 nm by the ELISA method using commercial kits according to the manufacturer’s instructions on the Bio-Tek ELX800 device. The results were divided by the amount of protein in the brain homogenates and expressed as amount/protein.

#### Histopathological examination

The brains taken from the animals were fixed by keeping them in 4% phosphate buffered paraformaldehyde (PFA) (pH 7.4) for 48 h. Afterwards the fixation process, routine histological follow-up procedures were performed according to our previous study (Ergul Erkec et al. 2024a). Paraffin-embedded tissues were cut at 5 μm thickness using a microtome and placed on poly-L-lysine slides. For general histological evaluations, sections were stained with hematoxylen&eosin (H&E) and LFB to detect myelin sheaths. LFB staining was carried out according to the manufacturer’s instructions with the Luxol Fast Blue Stain Kit. Subsequently, the sections were covered with entellan and evaluated under an Olympus BX53 microscope equipped with an Olympus DP74 camera attachment. An average of 8–10 sections of the sections were scored using the following evaluation standard (Qiu et al. 2018): For inflammation = 0: no inflammatory cells; 1: few scattered inflammatory cells; 2: inflammatory infiltrate around blood vessels; 3: large perivascular cuff appearance extending into the parenchyma. For demyelination: 0: absent; 1: rare foci; 2: few areas of demyelination; 3: large (confluent) areas of demyelination. Quantitative determination of myelinated areas after Luxol fast staining was performed by measuring the blue color intensity using Image-J software (Mojaverrostami et al. 2020).

### Statistical analysis

Descriptive Statistics for Y-Maze parameters are expressed as mean and standard error. Kruskal - Wallis test was used to compare groups in terms of these features. Statistical significance level was taken as 5% in the calculations and SPSS statistical package program (Version 23) was used for calculations. The Shapiro-Wilk test was used to determine whether the SOD, CAT, GSH-Px, MDA, IL- 6 and TNF-α data were normally distributed. Since the biochemical data in the groups were normally distributed, significant differences between the groups for the same parameter were determined with the One-Way ANOVA test. The Tukey HSD post-hoc test following ANOVA was executed to determine which group caused the differences. *p ≤ 0.05 is significant according to the other group. Statistical evaluations and graph plots of the histopathological results were made using GraphPad Prism 8.01 (GraphPad). The data were analysed using an unpaired t-test, and the significance levels were stated as *p* ≤ 0.05 *, *p* ≤ 0.01 **, *p* ≤ 0.001 ***, *p* ≤ 0.0001 ****. Results are presented as mean ± SEM (standard error of the mean).

#### The calculation for percentage of change for the measured biomarkers

The % change values of biochemical parameters according to the Control-S_42_ or Control mean values were calculated according to the formula below, respectively: [(Mean values of RM-Ghrelin groups* 100/Control-S_42_ mean value) − 100] and [(Mean values of RM-Ghrelin groups* 100/Control mean value) − 100].

The parameters in the histological changes (inflammation, demyelination, myelin staining intensity) were also calculated as percentage values. The obtained percentage data represent the percentage value of the average value of each parameter corresponding to the relevant group within the average values of all the groups. To calculate the percentage values, the formula (Bx100)/A was used, where A is the total sum of the average values of all the groups and B is the average value of the relevant group.

## Results

### Y maze test

The differences between the groups in terms of latency to exit from the starting arm, the number of arm entries, the number of successful changes and % spontaneous alternations were found insignificant (Table 1, *p* > 0.05).

**Table 1:** Alterations in Y-Maze parameters in control (Control_35_ and Control-S_42_) and treatment groups (demyelination, remyelination, remyelination + ghrelin 20 and remyelination + ghrelin 40) in a cuprizone-induced multiple sclerosis rat model

	DM (Mean ± SEM)	Control_35_ (Mean ± SEM)	RM (Mean ± SEM)	Control-S_42_ (Mean ± SEM)	RM-G20 (Mean ± SEM)	RM-G40 (Mean ± SEM)	*P* value
Latency to exit from the starting arm	14.00 ± 6.94	7.25 ± 2.71	7.14 ± 2.28	8.00 ± 2.79	6.50 ± 3.20	2.71 ± 0.36	0.052
The total number of arm entries	13.13 ± 1.11	11.63 ± 1.92	13.14 ± 1.44	12.29 ± 1.34	9.00 ± 2.92	8.71 ± 1.46	0.225
The number of successful changes	7.38 ± 0.63	6.25 ± 1.16	7.00 ± 1.48	6.57 ± 1.51	4.25 ± 2.29	4.57 ± 0.87	0.389
Spontaneous alternation (%)	68.88 ± 5.97	71.59 ± 9.38	59.57 ± 7.67	58.95 ± 8.90	64.17 ± 14.49	76.22 ± 9.22	0.623

### Inflammatory and OS parameters

**Fig. 2:**
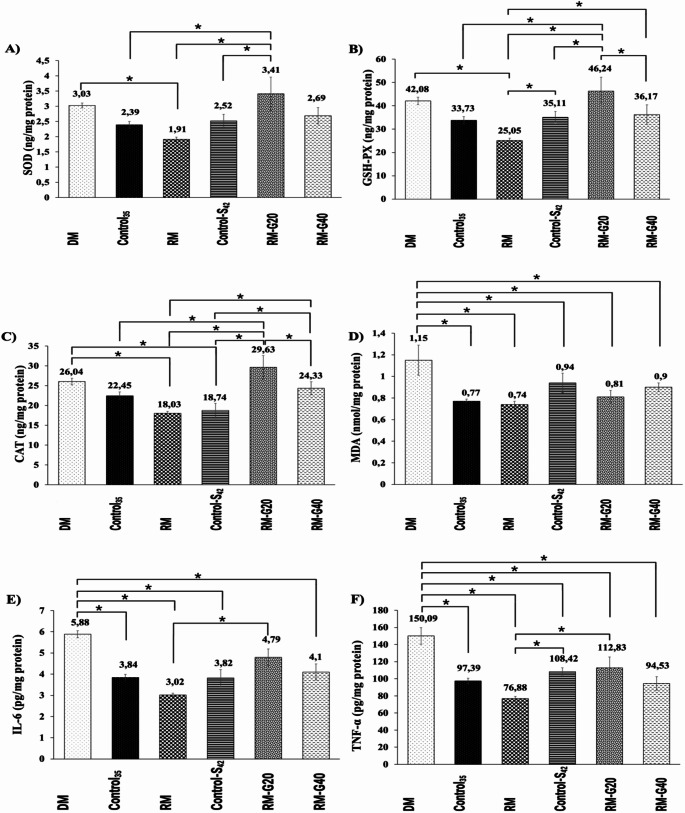
Alterations in inflammatory and oxidative stress parameters in control (Control_35_ and Control-Saline_42_) and treatment groups (demyelination, remyelination, remyelination + ghrelin 20 and remyelination + ghrelin 40) in a cuprizone-induced multiple sclerosis rat model (*n* = 8). **A**) SOD, **B**) GSH-Px, **C**) CAT, **(D)** MDA, **(E)** IL- 6, **(F)** TNF-α. *p ≤ 0.05 is significant according to the other group (Mean ± SEM). (DM: demyelination, RM: remyelination, G: ghrelin, S: saline, SOD: superoxide dismutase, GSH-Px: glutathione peroxidase, CAT: catalase, MDA: malondialdehyde, IL- 6: interleukin- 6, TNF-α: tumor necrosis factor-alpha)

An insignificant raising trend was found in SOD levels of DM compared to the Control_35_. CPZ cause an insignificant decrease trend in SOD levels of RM compared to the Control-S_42_. Ghrelin treatment significantly increased SOD levels at 20 µg/kg dose compared to the all the other groups except DM and RM-G40 (*p* = 0.008, Fig. 2A). GSH-Px levels of DM and Control_35_ groups were found similar. GSH-Px levels of RM group significantly decreased compared to the DM group (*p* = 0.001). GSH-Px levels of Control-S_42_ were found similar with DM and Control_35_ while they were found significantly increased compared to the RM group (*p* = 0.001). Ghrelin treatments significantly increased GSH-Px levels compared to the RM (*p* = 0.001, Fig. 2B). CAT levels of DM and Control_35_ groups were found similar. A significant decrease was found in the CAT levels of RM and Control-S_42_ compared to the DM. Ghrelin treatments significantly increased CAT levels compared to the RM and Control-S_42_ (*p* < 0.001, Fig. 2C). MDA levels were significantly increased in DM compared to the Control_35_ group (*p* = 0.003, Fig. 2D). MDA levels of Control_35_, RM, Control-S_42_, RM-G20 and RM-G40 were found similar (*p* = 0.003, Fig. 2D).

The highest levels of IL- 6 were found in DM. IL- 6 levels of RM and Control_35_ were significantly decreased compared to the DM (*p* < 0.001). IL- 6 levels of RM and Control-S_42_, Control_35_ and RM-G40 were found similar (*p* < 0.001). IL- 6 levels of RM-G20 group were significantly increased compared to the RM however they were still below to the those of DM (*p* < 0.001 Fig. 2E). TNF-α levels were significantly increased in DM compared to the Control_35_ (*p* < 0.001). They were significantly decreased in RM, Control-S_42_, RM-G20 and RM-G40 compared to the DM (*p* < 0.001). However, TNF-α levels of the Control-S_42_ and RM-G20 were found significantly elevated compared to the RM. The lowest TNF-α levels were belonged to RM and the TNF-α levels of Control_35_ and RM-G40 were found similar to those of RM (*p* < 0.001, Fig. 2F).

**Fig. 3:**
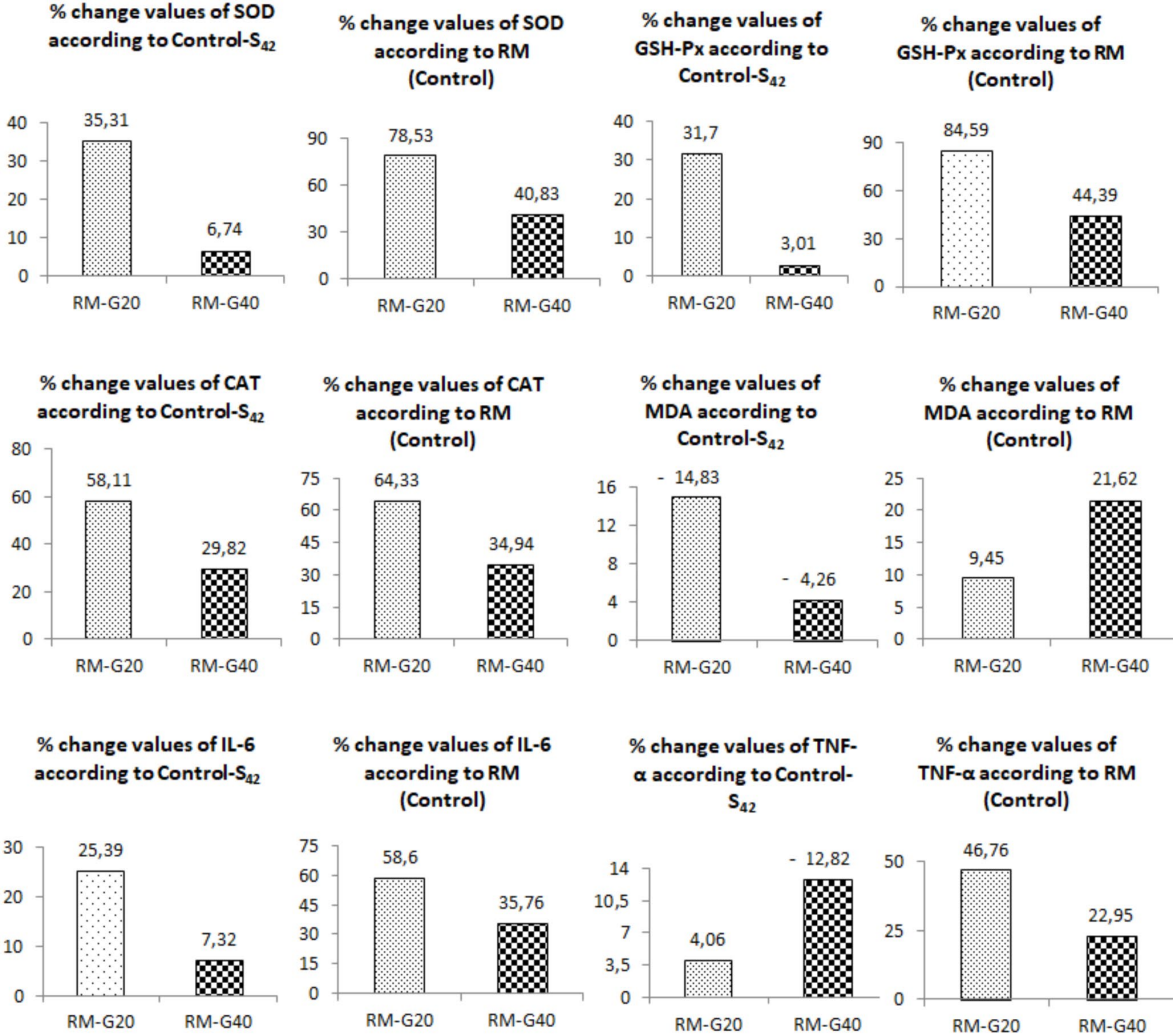
Percentages (%) of change ​​in biochemical parameters in ghrelin treated groups (RM-G20 and RM-G40), according to RM (control) and Control-S_42_ groups (*n* = 8). (RM: remyelination, G: ghrelin, S: saline, SOD: superoxide dismutase, GSH-Px: glutathione peroxidase, CAT: catalase, MDA: malondialdehyde, IL- 6: interleukin-* 6*, TNF-α: tumor necrosis factor-alpha)

When oxidative stress and inflammatory parameters were taken into consideration, the percentage change values of the groups receiving Ghrelin treatment (RM-G20 and RM-G40) compared to the Control-S_42_ (as negative control) group were presented in Fig. 3.

### Histopathological results

**Fig. 4:**
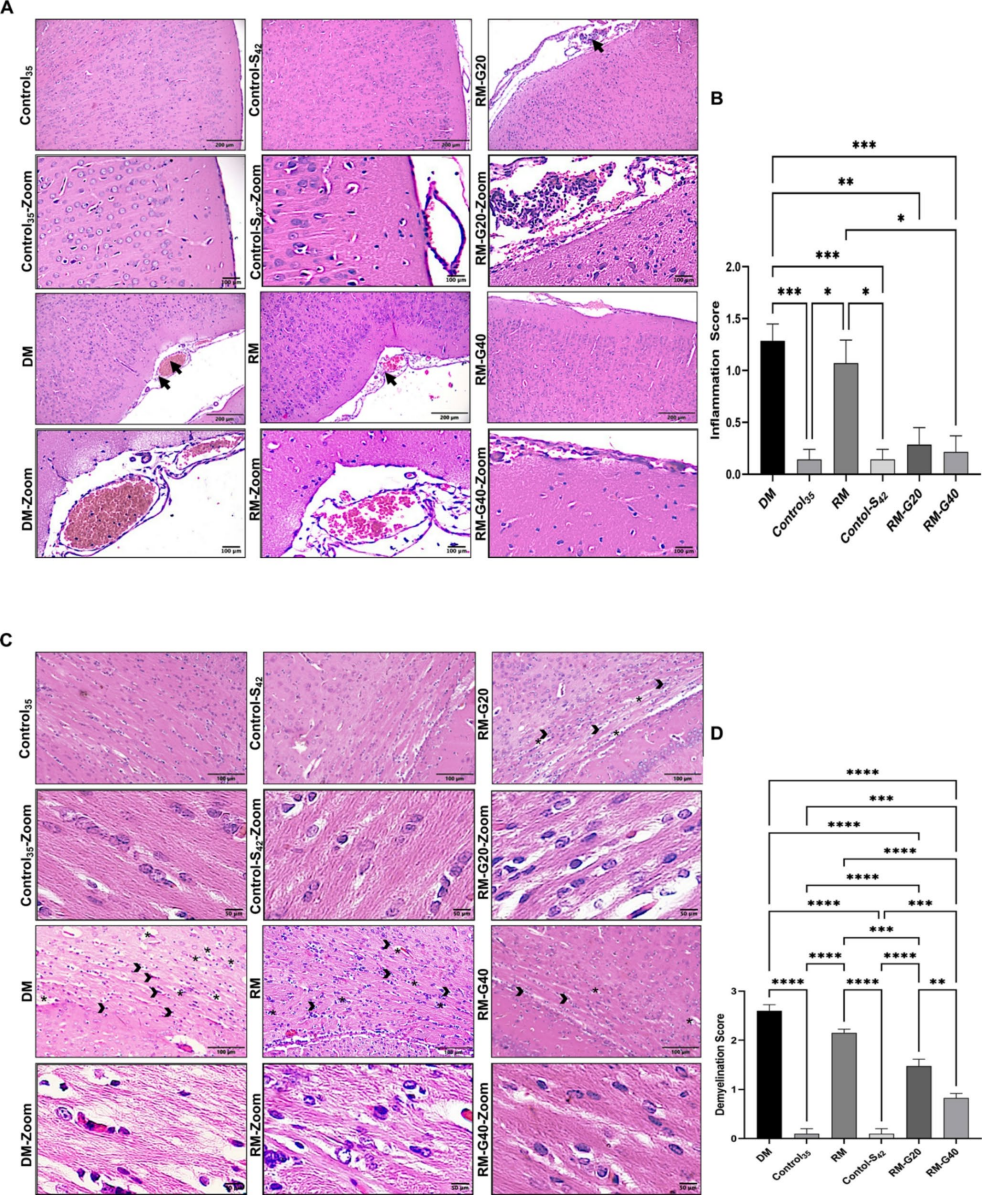
Histological alterations in control (Control_35_ and Control-Saline_42_) and treatment groups (demyelination, remyelination, remyelination + ghrelin 20 and remyelination + ghrelin 40) in a cuprizone-induced multiple sclerosis rat model (*n* = 8). (**A**) General histological organization of brain cortical areas. Black arrows indicate lymphatic infiltration (Scale bar: 200 μm and 100 μm). (**B**) Bar graph of inflammation score. (**C**) Arrangement of nerve bundles in the corpus callosum. Black arrowheads indicate disorganized and fragmented demyelinated nerve fibers, and asterisks indicate areas of vacuolization (Scale bar: 100 μm and 50 μm). (**D**) Bar graph of demyelination score. (*p* ≤ 0.05 *; *p* ≤ 0.01 **; *p* ≤ 0.001 ***; *p* ≤ 0.0001 ****) (DM: demyelination, RM: remyelination, G: ghrelin)

Histopathological characteristics, considered the gold standard for revealing MS-related tissue damage, typically include immune cell infiltration, demyelination, and axonal damage (Brück 2007). It is critical to demonstrate the changes appearing at the tissue and cell level in MS diagnosis and treatment models. Within the scope of this study, a combination of histological procedures was performed to determine inflammatory areas compatible with MS and to demonstrate myelin damage and repair. Evaluation of the general histological organization, lymphatic infiltration, demyelination and degeneration in the brain tissue including cortical areas and corpus callosum was performed with H&E staining. Control groups (Control_35_ and Control-S_42_) were found to exhibit a normal histological organization (Fig. 4). Lymphatic infiltration was apparent in cortical areas, with an increase in vascularization in the CPZ-induced groups (DM and RM) (Fig. 4A). Significant increases in lymphatic cells were observed in DM (*p* ≤ 0.001) and RM (*p* ≤ 0.05) compared to the control groups (Fig. 4B). When the inflammation score was evaluated as a percentage (%), the average was 41%, 5%, 33%, 5%, 9%, and 7% for DM, Control_35_, RM, Control-S_42_, RM-G20, and RM-G40, respectively. Furthermore, treatment groups exhibited a significant decrease in lymphatic infiltration areas (*p* ≤ 0.05) (Fig. 4A and B).

Light microscope examinations of the corpus callosum areas stained with H&E were examined to detect changes in myelin content. As a result of H&E staining, the presence of large vacuolation areas and irregular, fragmented, demyelinating nerve fibers were detected in CPZ-treated groups (Fig. 4C). Furthermore, the presence of oligodendrocytes characterized by pyknotic nuclei was observed in demyelinated areas (Fig. 4C). The CPZ-treated groups exhibited significantly higher levels of demyelination compared the control groups (*p* ≤ 0.0001). Ghrelin-treated groups displayed a considerable reduction in disordered and fragmented demyelinated nerve bundles, as well as vacuolization (Fig. 4C and D) (*p* < 0.05). Furthermore, the RM-G40 group exhibited a significantly decreased level of demyelinated areas than the RM-G20 group (*p* ≤ 0.01). When the demyelination score was evaluated as a percentage (%), the average is 36%, 1%, 30%, 2%, 20% and 11% for DM, Control_35_, RM, Control-S_42_, RM-G20 and RM-G40, respectively.

**Fig. 5:**
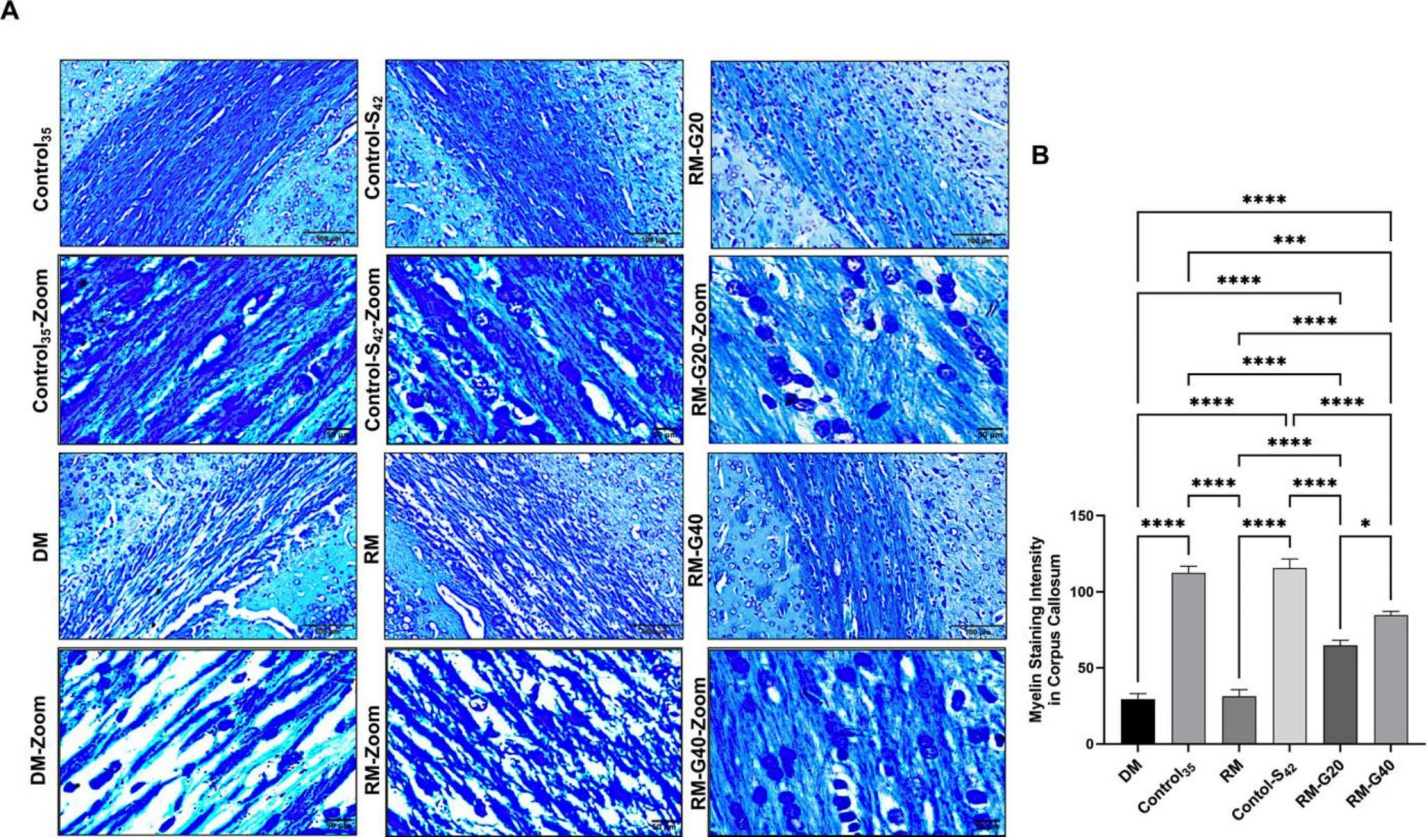
Alterations in myelin content in control (Control_35_ and Control-Saline_42_) and treatment groups (demyelination, remyelination, remyelination + ghrelin 20 and remyelination + ghrelin 40) in a cuprizone-induced multiple sclerosis rat model (*n* = 8). (**A**) Myelin content (blue) of corpus callosum was visualized using LFB staining. (Scale bar: 100 μm and 50 μm). (**B**) Bar graph of myelin staining intensity in the corpus callosum (*p* ≤ 0.05 *; *p* ≤ 0.01 **; *p* ≤ 0.001 ***; *p* ≤ 0.0001 ****) (DM: demyelination, RM: remyelination, G: ghrelin)

To confirm the findings of myelin changes seen as a result of H&E staining, the LFB staining method, which is frequently used to show myelination levels in tissues, was used (Carriel et al. 2017). Based on the LFB staining results, the myelin content in the corpus callosum regions was measured and evaluated with the Image-J program. Our results displayed that myelin content was significantly reduced in the DM and RM groups compared to the control groups (*p* ≤ 0.0001) (Fig. 5A and B). Compared to the RM group, myelin content was found to be higher in the RM-G20 (*p* ≤ 0.0001) and RM-G40 (*p* ≤ 0.0001) groups (Fig. 5A and B). The comparison of groups treated with ghrelin revealed that myelin content increased as the dose increased (*p* ≤ 0.05, Fig. 5B). According to the LFB staining pattern, when the myelin intensity score was evaluated as a percentage (%), the average was 8%, 25%, 7%, 26%, 15% and 19% for DM, Control_35_, RM, Control-S_42_, RM-G20 and RM-G40, respectively.

## Discussion

In the present study Y-maze test is used for assessment of working memory and cognitive function. In a previous study conducted on adult rats administered 0.4% CPZ for 5 weeks, it was reported that there was no significant difference in the total number of arm entries in the Y maze between the experimental groups, while a significant decrease in working memory was reported in the CPZ-administered group (Omotoso et al. 2018). A study conducted on adult rats using a 0.5% Cuprizone diet for 45 days reported that the percentage correct alternation was significantly decreased in the CPZ only treated group compared to the control group (Ogunlade et al. 2021). In the present study, Y-maze test results revealed that 2 weeks 0.6% cuprizone exposure does not affect latency to exit the start arm, total number of arm entries and % spontaneous alternation which suggests that CPZ administration did not cause a difference in the working memory and cognitive function of the rats. It might be suggested that the effects of cuprizone on working memory and cognitive function in rats, could alter by the dose of cuprizone, the exposure time and the age of the rats.

MS, a chronic inflammatory CNS disorder, is one of the most prevalent reason of disability in young adults (Filippi et al. 2018), whose exact cause is unknown, prognosis is unpredictable, and treatment options are very limited (Ostojic 2022). Overproduction of pro-inflammatory cytokines has a role in the inflammatory process seen in MS (Das 2012). Our results revealed that CPZ administration led to a significant increase in proinflammatory cytokines (IL- 6 and TNF-α) in DM group. Similar to our findings, previous studies reported significantly elevated TNF-α or TNF-α mRNA levels (Huang et al. 1999; Mikova et al. 2001) and IL- 6 levels in MS patients compared to the healthy controls (Eslami et al. 2003). A close relationship between inflammation and neurodegeneration was reported in the illness phases of MS (Frischer et al. 2009). In our study, in addition to the increase in pro-inflammatory cytokine levels due to CPZ administration, a significant increase in the number of lymphatic cells and inflammatory cell, and large vacuolation areas were detected. Also, ghrelin treatments significantly decreased TNF-α and IL- 6 levels compared to the DM group. This result indicated that ghrelin had an anti-inflammatory function by suppressing proinflammatory cytokines such as TNF-α and IL- 6. Consistent with this result, a previous study reported that the anti-inflammatory properties of ghrelin may be due to its ability to suppress inflammatory cytokine release and inflammatory microglial activation (Jiao et al. 2017).

While inflammation is known to be an important factor in MS pathology, OS contributes to the tissue damage and increases the existent inflammatory response (Pegoretti et al. 2020). It was reported that inflammation associated OS exacerbates the functional outcome of the MS, enhances neuronal injury, and may increase the rate of disease progression (Padureanu et al. 2019). In a previous study it was reported that SOD activity did not change however a significant decrease in GSH-Px and an increase in CAT activity were found in MS patients compared to the healthy group (Bizoń et al. 2023). Similarly in this study SOD levels did not change between DM and Control_35_ or RM and Control-S_42_ groups. In addition, the difference between CAT levels of the DM and Control_35_ or RM and Control-S_42_ groups was found insignificant. However, GSH-Px levels of RM were significantly decreased compared to the Control-S_42_.

Antioxidative properties of ghrelin reported in various neurological disorders: It was reported that ghrelin was suppressed OS and ROS formation in a Parkinson’s disease model (Wang et al. 2020). A previous study reported that ghrelin decreased OS damage and neuronal apoptosis after hypoxia-ischemia in rats (Huang et al. 2019). Similarly, in the present study ghrelin treatment at 20 µg/kg dose significantly increased SOD, GSH-Px and CAT levels compared to the RM. Ghrelin treatment at 40 µg/kg dose significantly increased GSH-Px and CAT levels compared to the RM group. Ghrelin showed antioxidant effects at both doses used in this study, but the 20 µg/kg dose of ghrelin was found to be more effective than the 40 µg/kg dose in terms of CAT and GSH-Px levels. It was reported that ghrelin inhibits microglial activation (Moon et al. 2009; Jiao et al. 2017) and, upregulating the expression of mitochondrial uncoupling protein- 2 (UCP2) (Huang et al. 2019). UCP2 is involved in the inhibition of apoptotic factors and oxidative stress (Dutra et al. 2018). Therefore, it may be suggested that the antioxidant and neuroprotective effects of ghrelin in CPZ-induced MS model could be derived from its inhibitory effects on mitochondrial inflammation activity and ROS production.

Free radicals lead to oxidative modification of lipids and the initiation of lipid peroxidation, which leads to the destruction of lipid-rich regions such as the myelin sheath (Ortiz et al. 2013) and the formation of reactive aldehydes such as MDA (Esterbauer et al. 1991; Ortiz et al. 2013). An increase in lipid peroxidation is inversely proportional to antioxidant balance and is a crucial criterion for OS (Sarlaki et al. 2022). In this study MDA levels were evaluated as a biomarker of lipid peroxidation and it was found that MDA levels were significantly increased in demyelination group. Similarly with our results in a previous study MDA levels were reported to be significantly increased in MS patients than those in Controls (Ghonimi et al. 2021). In a previous study, ghrelin treatment was reported to decrease the MDA levels in a rat model of Alzheimer’s disease (Sarlaki et al. 2022). Similarly, our data revealed that MDA levels were significantly decreased with ghrelin treatments (at both doses). This effect may be derived from ghrelin’s inhibitory effects on ROS production. In a previous study it was suggested that lipid peroxidation involved to the development of myelin loss and neurodegeneration in MS (Wang et al. 2014). Similarly, in this study, due to CPZ administration, in addition to increased MDA levels, a significant decrease in myelin content and irregular, fragmented, demyelinating nerve fibers were detected. In previous experimental autoimmune encephalomyelitis studies, it was reported that ghrelin was reduced demyelination (Souza-Moreira et al. 2013; Liu et al. 2019). Similarly, in the present study it was found that ghrelin reduced demyelinating areas and increased myelin content also in a CPZ-induced MS model in rats. It was observed that ghrelin significantly reduced demyelination and inflammation scores and increased myelin content at both doses. While these effects were equal in both doses in terms of reducing inflammatory scores, ghrelin was found to be more effective at the dose of 40 µg/kg compared to the dose of 20 µg/kg in terms of increasing myelin content and reducing demyelination. Microglia are involved in MS pathology as triggers of inflammation at all phases of lesion formation (Guerrero and Sicotte 2020). It is known that myelin is damaged and axonal and synaptic activity is impaired by chronic microglial inflammatory activity in both clinical MS and MS animal models (Distéfano-Gagné et al. 2023). Ghrelin treatment is reported to inhibit the activation of microglia (Moon et al. 2009). It was also reported that MDA levels were increased in MS patients who taking no disease modifying therapy than those who taking interferon-β (Ghonimi et al. 2021). Neuronal interferon-β has been reported to be essential for mitochondrial homeostasis and metabolism and prevents excessive ROS (Tresse et al. 2021). It was reviewed that ghrelin increases neuronal survival due to its apoptosis, inflammation and OS reducing and mitochondrial function improving actions (Jiao et al. 2017). Therefore, the reason for the myelin loss-reducing effect of ghrelin may be due to its suppression of MDA levels in the cuprizone-induced MS model and it has antioxidant, ROS-reducing and mitochondrial dysfunction-regulating effects.

## Conclusion

In conclusion, our data clearly revealed that CPZ causes a significant decrease in antioxidant GSH-Px levels and myelin content and a significant increase in lipid peroxidation, proinflammatory cytokine (TNF-ɑ, IL- 6) levels, inflammation score, demyelination score, the number of lymphatic cells and inflammatory cells. However, ghrelin has antioxidant (an increasing in SOD, GSH-Px and CAT levels), anti-inflammatory (a decreasing TNF-ɑ and IL- 6 levels according to the DM group), and neuroprotective (myelin loss-reducing and myelin content protective) effects in CPZ-induced MS model in rats which were reflected by increased antioxidant levels, decreased proinflammatory cytokine levels and remyelination. In addition, it could be suggested that the 20 µg/kg Ghrelin dose is more effective than the 40 µg/kg Ghrelin dose on the increase in antioxidant enzyme levels and the decrease in lipid peroxidation levels when the percentage changes according to the Control-S_42_ and RM were evaluated. Our results may suggest that ghrelin might be a hopeful candidate agent in MS treatment. However, signaling pathways leading to the neuroprotective effects of ghrelin and gender-related variables were not investigated in this study. Further preclinical studies are needed to investigate the relevant signaling pathways leading to the neuroprotective effects of ghrelin and gender-related variables in the cuprizone-induced MS model.

## Data Availability

The datasets generated during and/or analyzed during the current study are available from the corresponding author on reasonable request.
